# Supplementary material for: Cross-plane coherent acoustic phonons in two-dimensional organic-inorganic hybrid perovskites
Source: Nat Commun. 2018 May 22;9:2019. doi: 10.1038/s41467-018-04429-9 (PMC5964251; doi:10.1038/s41467-018-04429-9)
Supplement: Supplementary file 1 — Supplementary Information [file 41467_2018_4429_MOESM1_ESM.pdf]

Cross-plane coherent acoustic phonons in two-dimensional organic-inorganic hybrid perovskites

P. Guo et al.

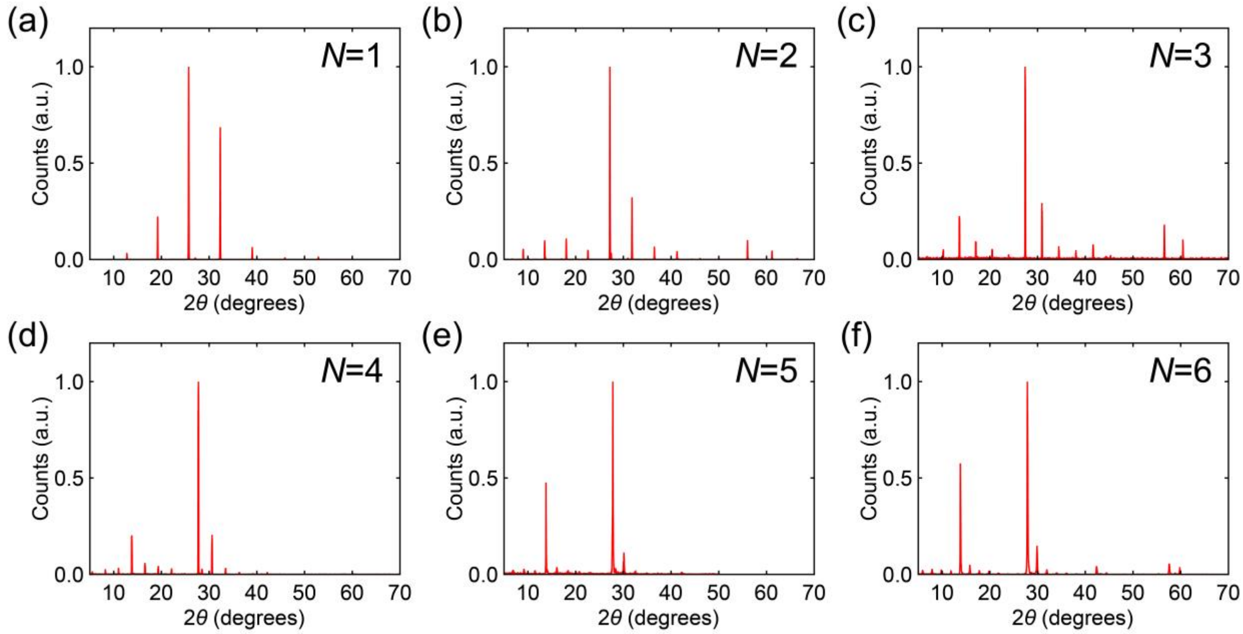

Supplementary Figure 1. Specular *θ*-2*θ* X-ray diffraction patterns of the single-crystal flakes (*N*=1 to *N*=6).

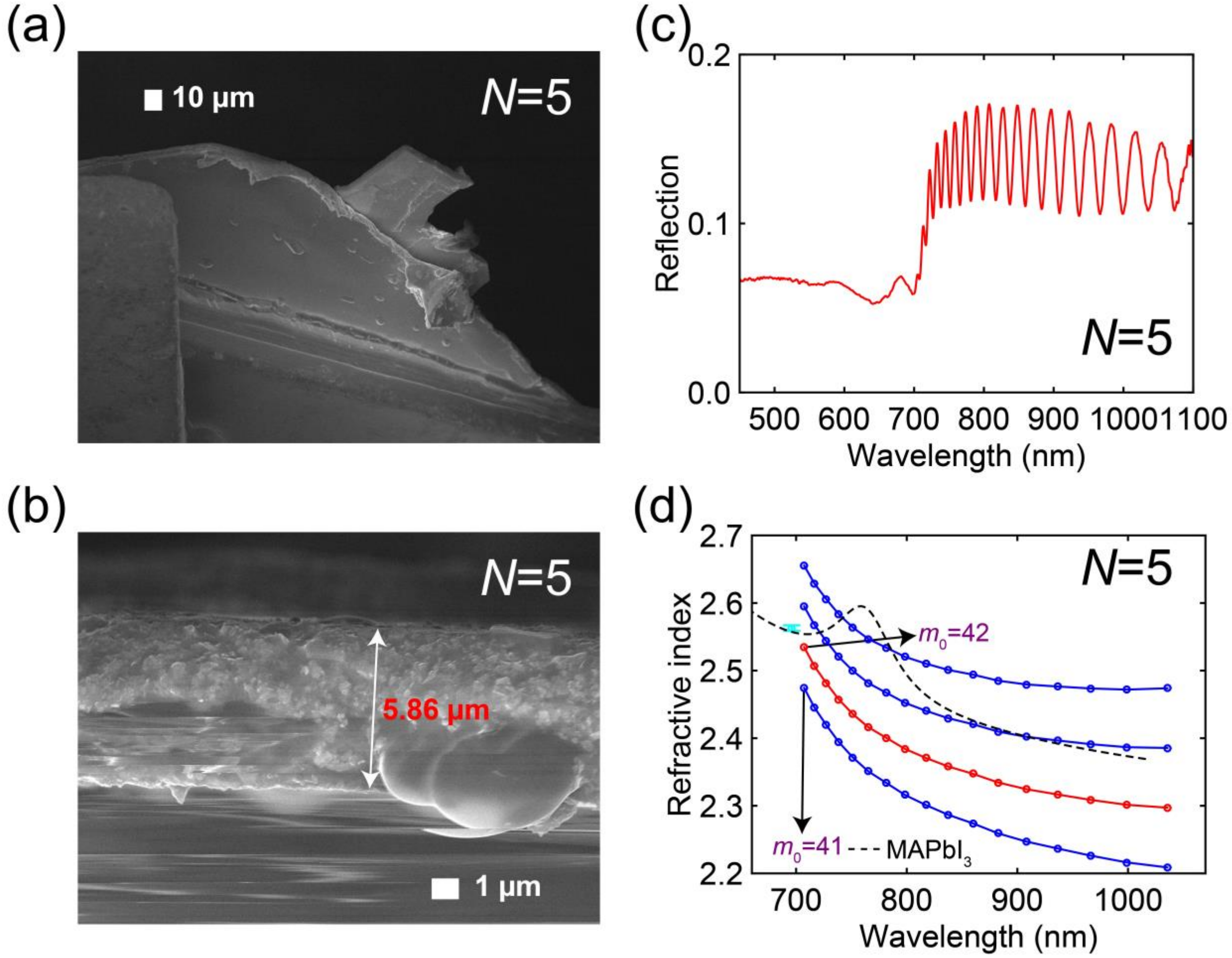

Supplementary Figure 2. Quantification of the refractive index for *N*=5. (a) Angular view and (b) Side-view of the examined flake taken under SEM. (c) Reflection spectrum and (d) Several possible $n'(\lambda)$ curves obtained using various mode numbers, with the correct $n'(\lambda)$ curve shown in red. The cyan dot denotes the refractive index of *N*=5 at the absorption edge determined from step 2.

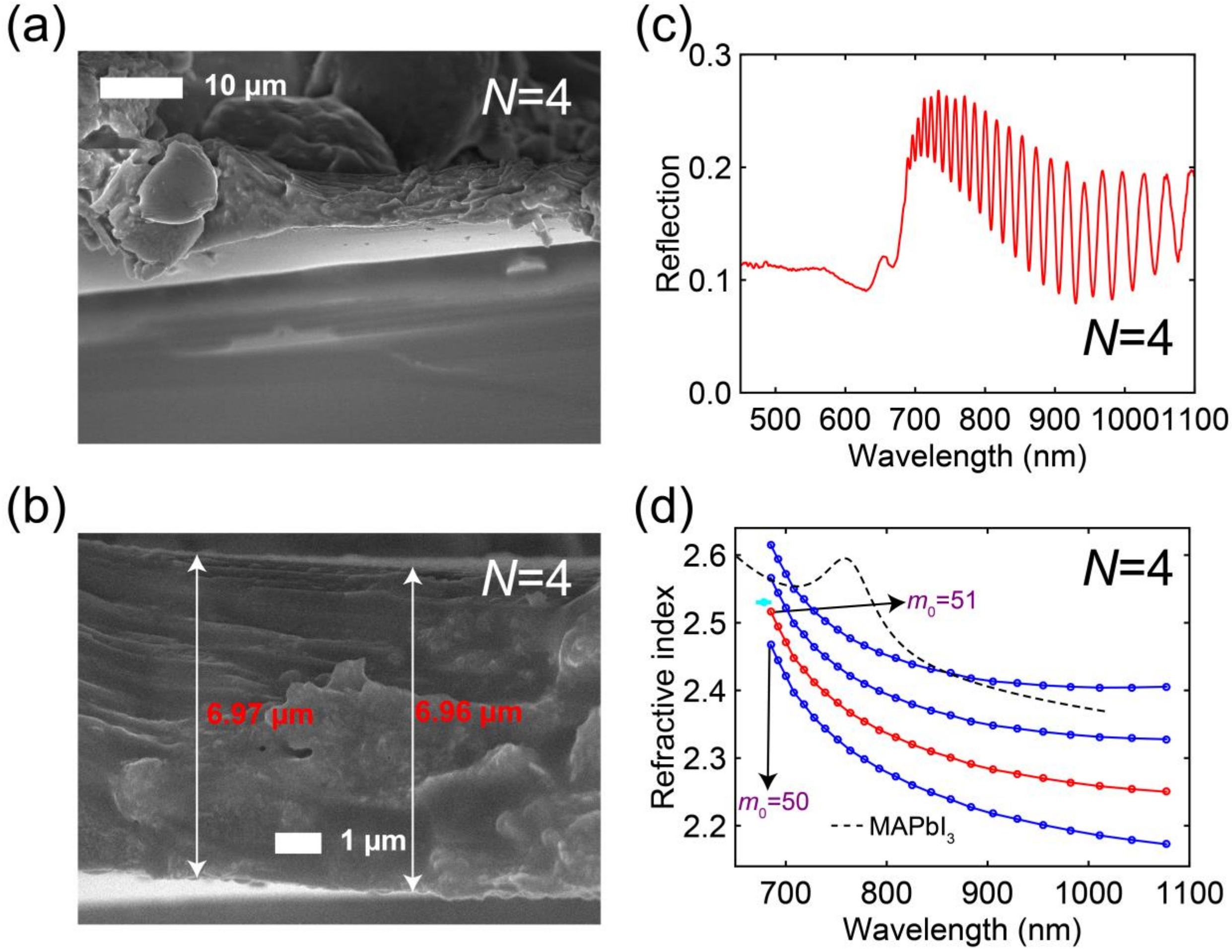

Supplementary Figure 3. Quantification of the refractive index for *N*=4. (a) Angular view and (b) Side-view of the examined flake taken under SEM. (c) Reflection spectrum and (d) Several possible $n'(\lambda)$ curves obtained using various mode numbers, with the correct $n'(\lambda)$ curve shown in red. The cyan dot denotes the refractive index of N=4 at the absorption edge determined from step 2.

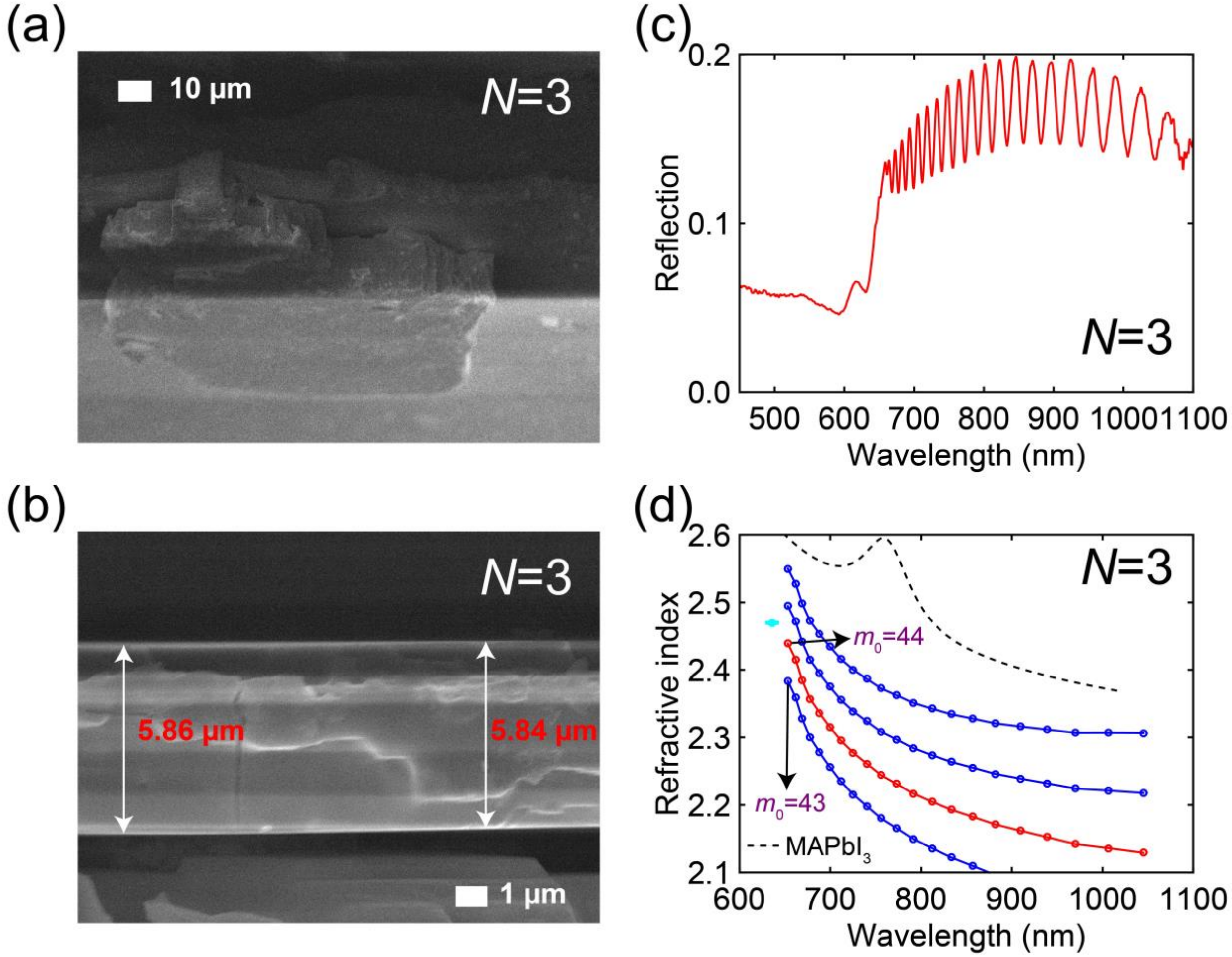

Supplementary Figure 4. Quantification of the refractive index for *N*=3. (a) Angular view and (b) Side-view of the examined flake taken under SEM. (c) Reflection spectrum and (d) Several possible $n'(\lambda)$ curves obtained using various mode numbers, with the correct $n'(\lambda)$ curve shown in red. The cyan dot denotes the refractive index of *N*=3 at the absorption edge determined from step 2.

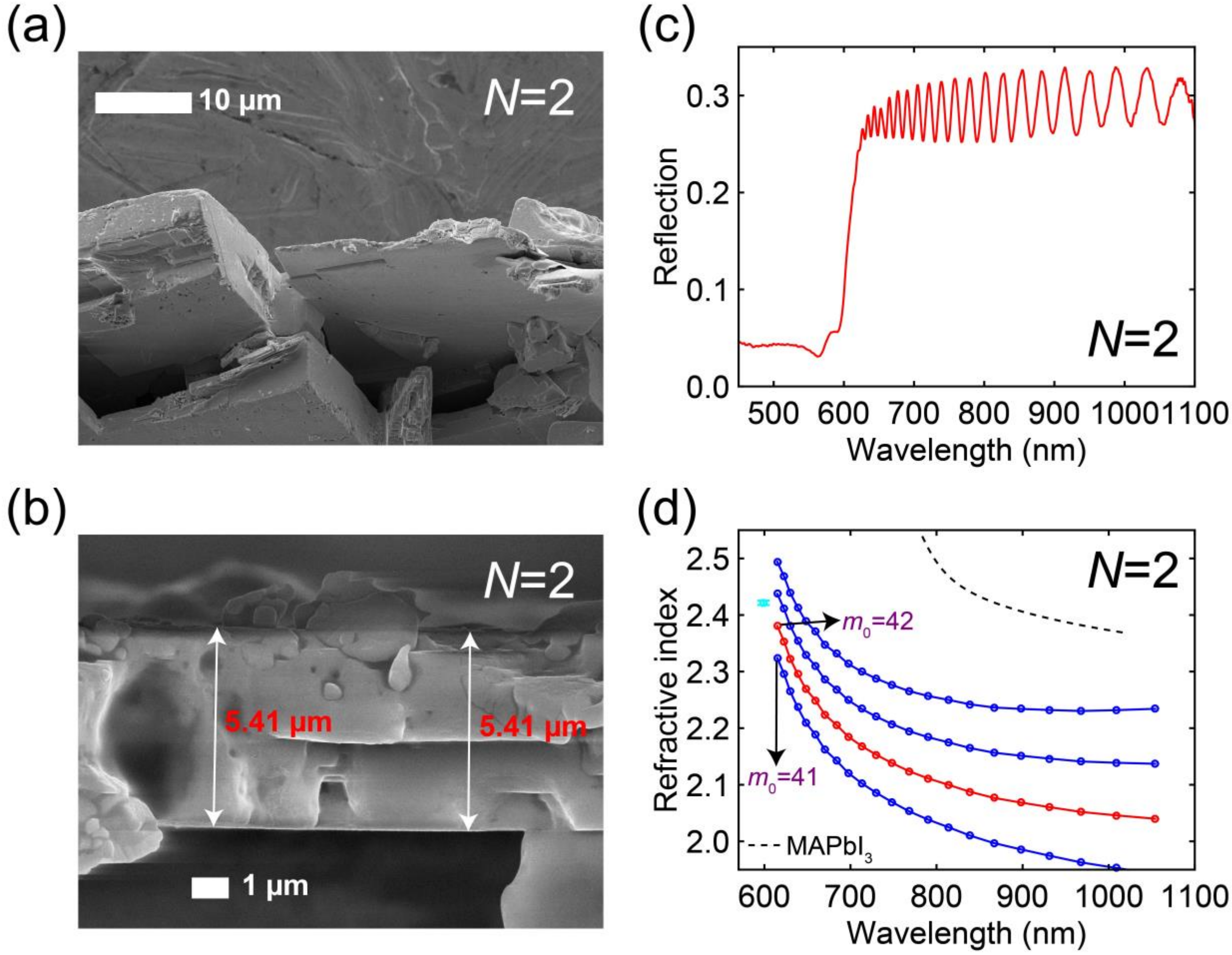

Supplementary Figure 5. Quantification of the refractive index for *N*=2. (a) Angular view and (b) Side-view of the examined flake taken under SEM. (c) Reflection spectrum and (d) Several possible $n'(\lambda)$ curves obtained using various mode numbers, with the correct $n'(\lambda)$ curve shown in red. The cyan dot denotes the refractive index of N=2 at the absorption edge determined from step 2.

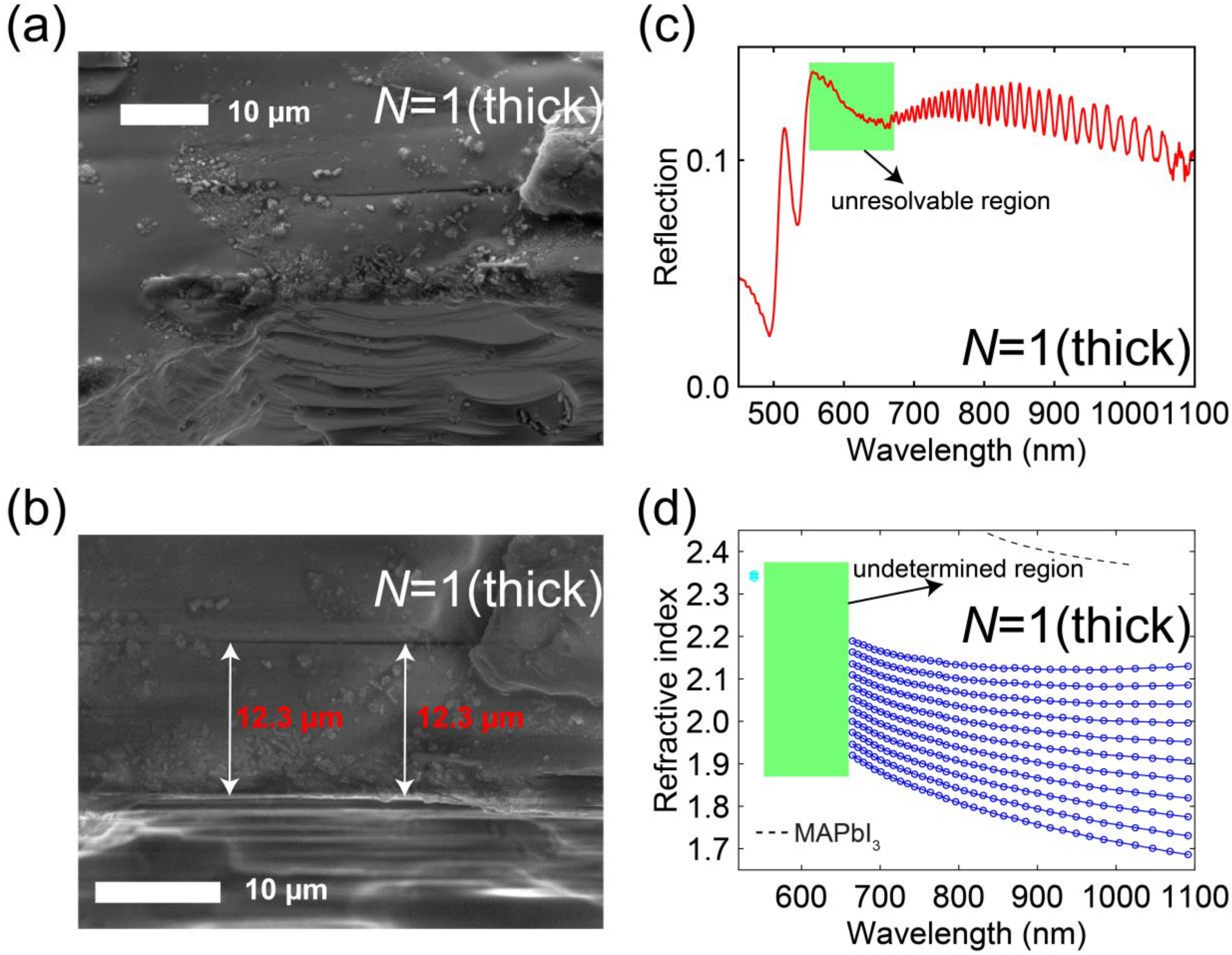

Supplementary Figure 6. Quantification of the refractive index for *N*=1, part 1. (a) Angular view and (b) Side-view of the thick layer due to a fissure formed at the edge of an *N*=1 flake. (c) Reflection spectrum and (d) Several possible $n'(\lambda)$ curves obtained using various mode numbers for the thick layer. The cyan dot denotes the refractive index of *N*=1 at the absorption edge determined from step 2.

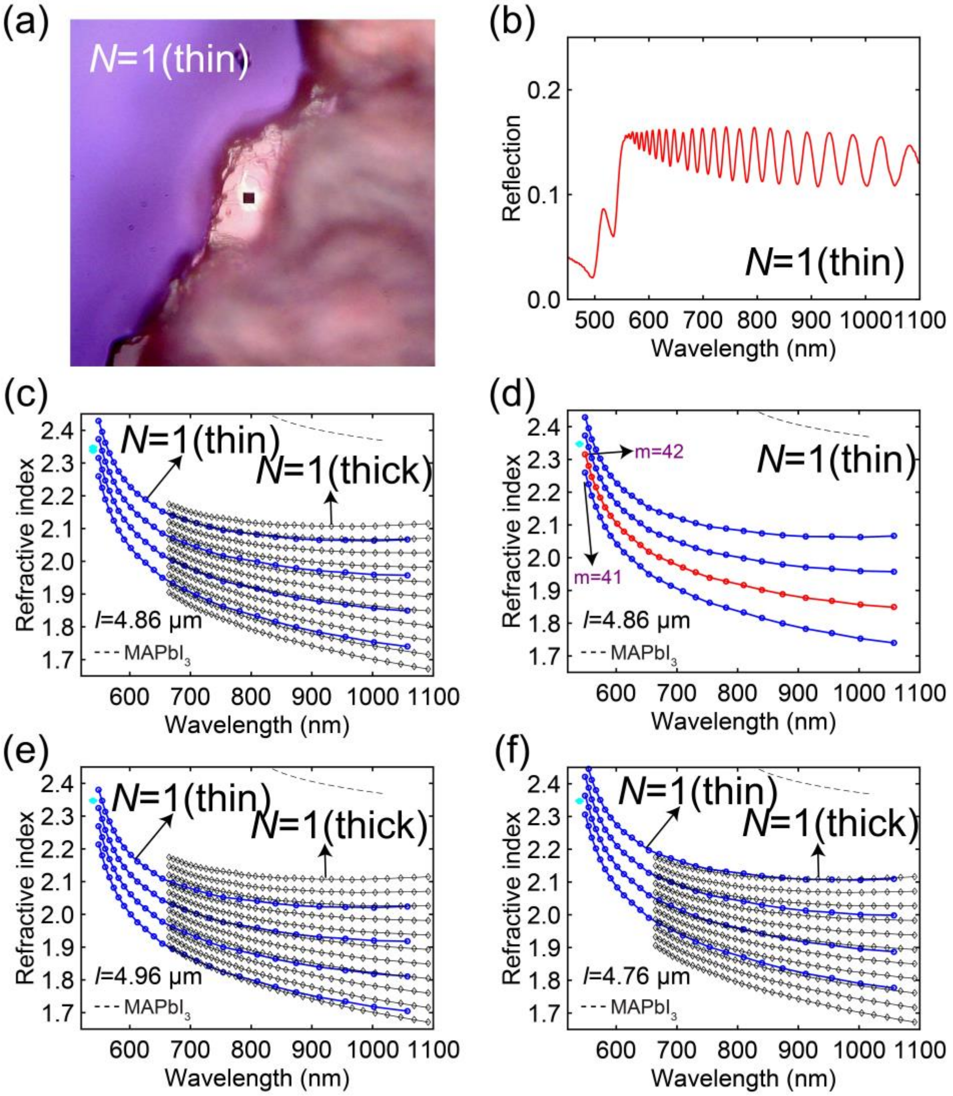

Supplementary Figure 7. Quantification of the refractive index for *N*=1, part 2. (a) Optical microscopic image and (b) Reflection spectrum of the thin layer of *N*=1. (c) Several possible $n'(\lambda)$ curves obtained using various mode numbers for the thin layer, whose dispersions match the $n'(\lambda)$ curves obtained using various mode numbers for the thick layer (which also appears in Supplementary Fig. 6d). The determined thickness for the thin layer is 4.86 μm. (d) Several possible $n'(\lambda)$ curves obtained using various mode numbers for the thin layer with the correct one shown in red. (e) and (f) present the $n'(\lambda)$ curves obtained using incorrect thicknesses for the thin layer; 4.96 μm shown in (e) and 4.76 μm shown in (f). The cyan dots in (c) to (f) denote the refractive index of *N*=1 at the absorption edge determined from step 2.

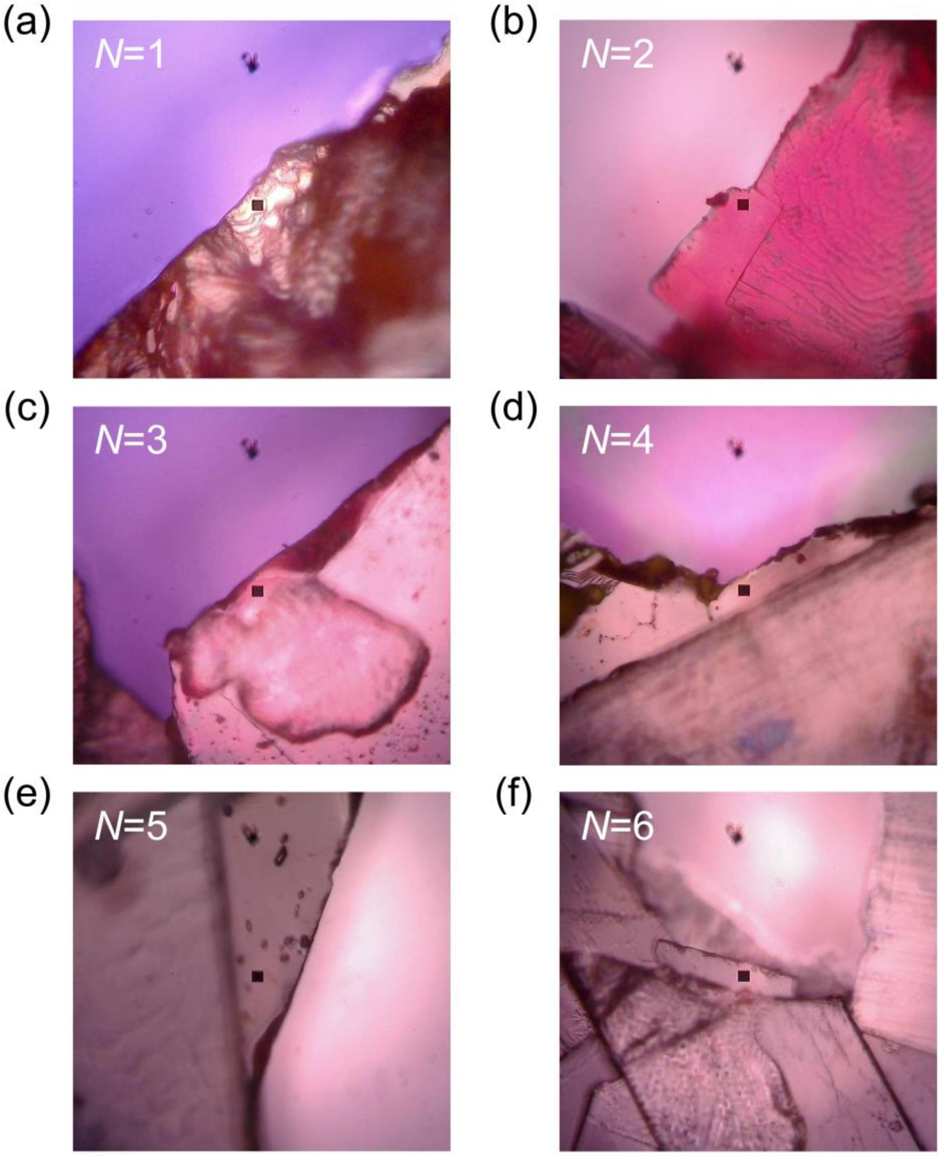

Supplementary Figure 8. Optical microscopic images of the small flakes used for step 1 (captured during the reflection measurements). The black-square dot highlights the location at which the reflection spectrum was taken.

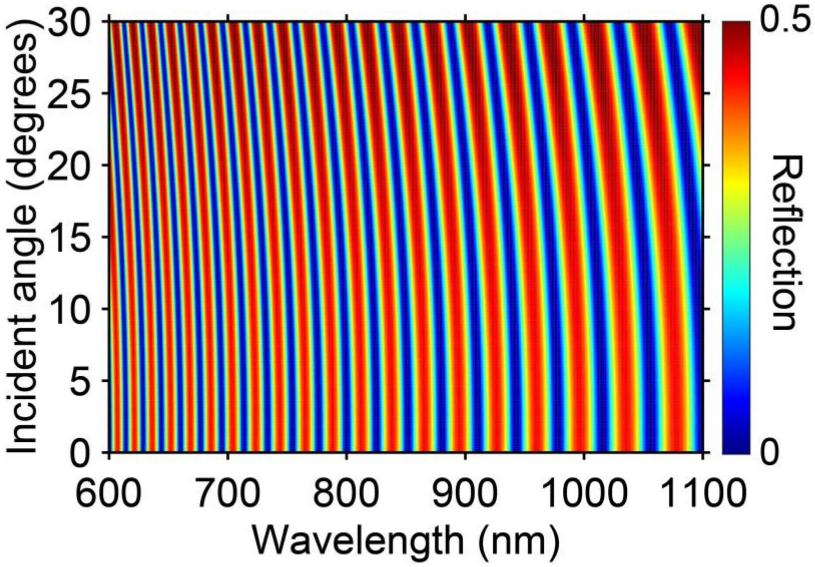

Supplementary Figure 9. Reflection of a dielectric layer floating in air at various incidence angles and wavelengths obtained from transfer matrix calculation. The dielectric layer is 6-μm thick, having a constant real part of refractive index of 2.2, and zero imaginary part.

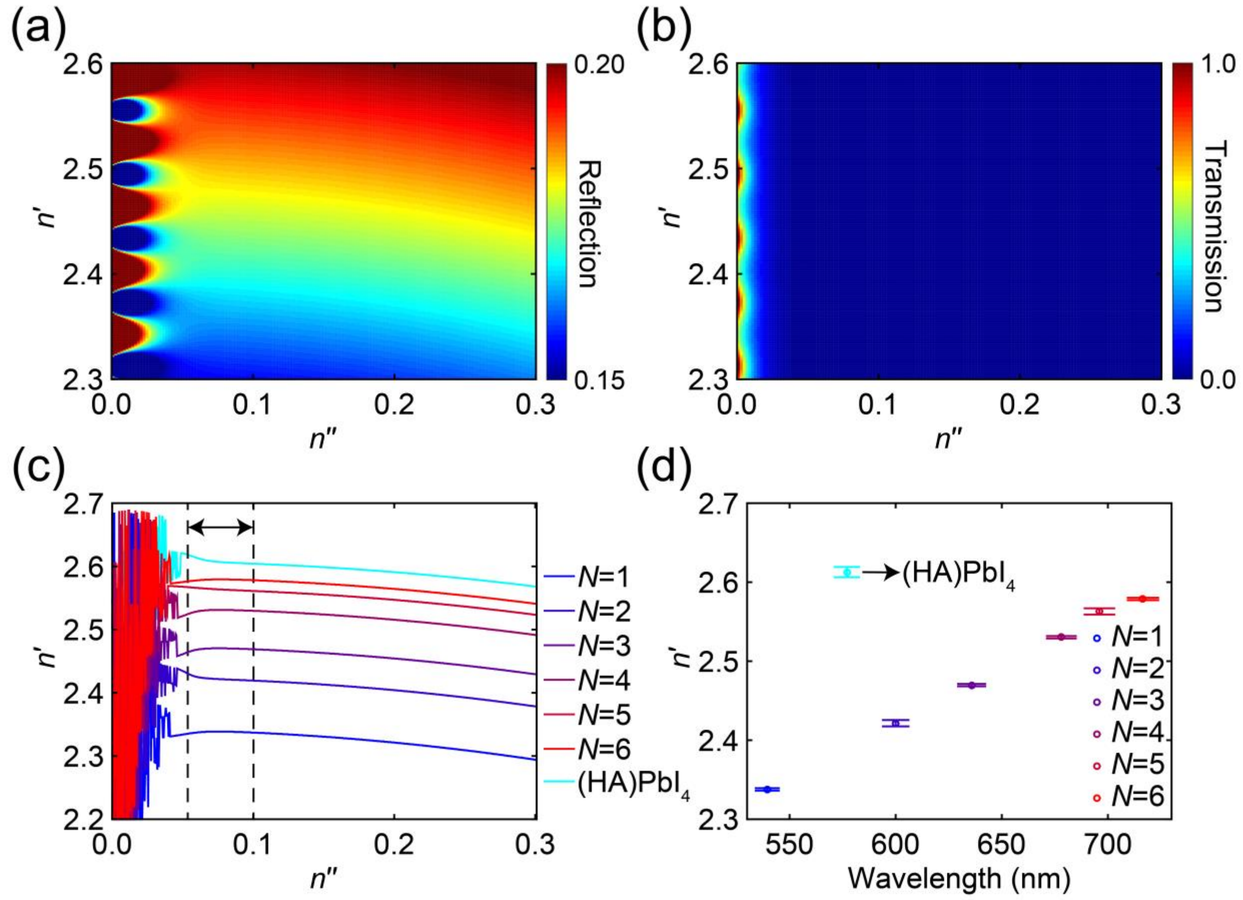

Supplementary Figure 10. Determination of the refractive indices at the absorption edge wavelengths. Transfer-matrix calculation predicted reflection in (a), and transmission in (b), for a 6-μm thick slab floating in air. The slab takes various values of the real (*n*′) and imaginary parts (*n*″) of refractive index, shown as the *y* and *x* axes, and the reflection & transmission are color-coded in (a) and (b), respectively. (c) The values of *n*′ as a function of *n*″, which gives a calculated reflection matching the measured one (shown in Supplementary Fig. 11) at the absorption edge wavelengths. (d) The identified values of *n*′ plotted at the corresponding absorption edge wavelengths for different compositions examined in the work.

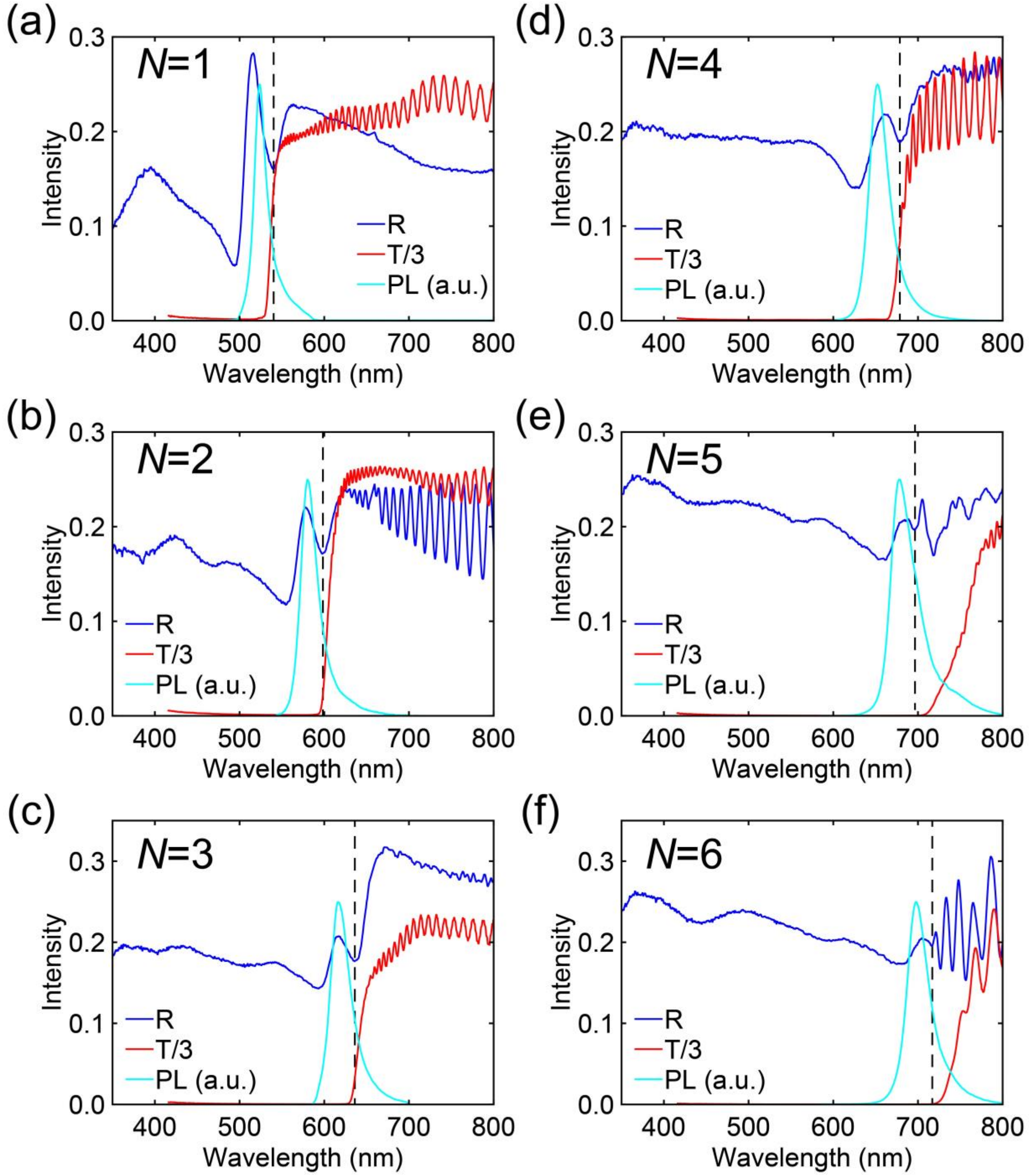

Supplementary Figure 11. Reflection (R), transmission (T) and photoluminescence (PL) spectra of *N*=1 to 6. The PL spectra were acquired by excitation at 450 nm. Note that the transmission and reflection spectra for each composition were not taken at the same spot, and the transmission values are divided by a factor of 3.

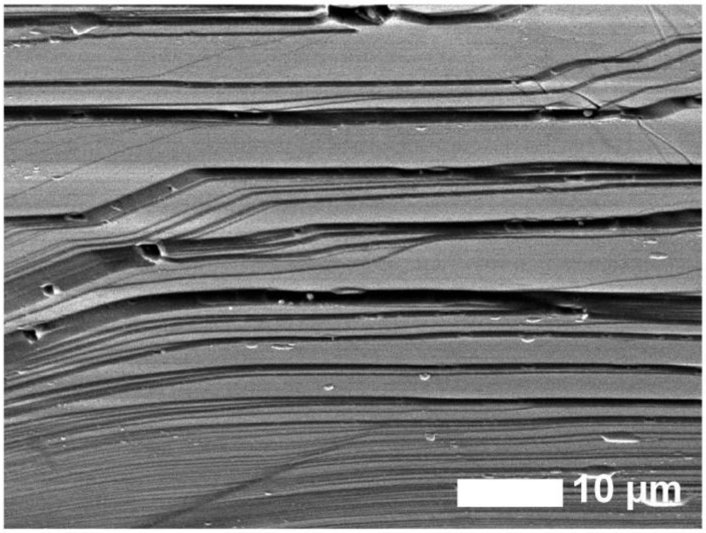

Supplementary Figure 12. SEM image of an *N*=5 flake demonstrating the layer-by-layer growth.

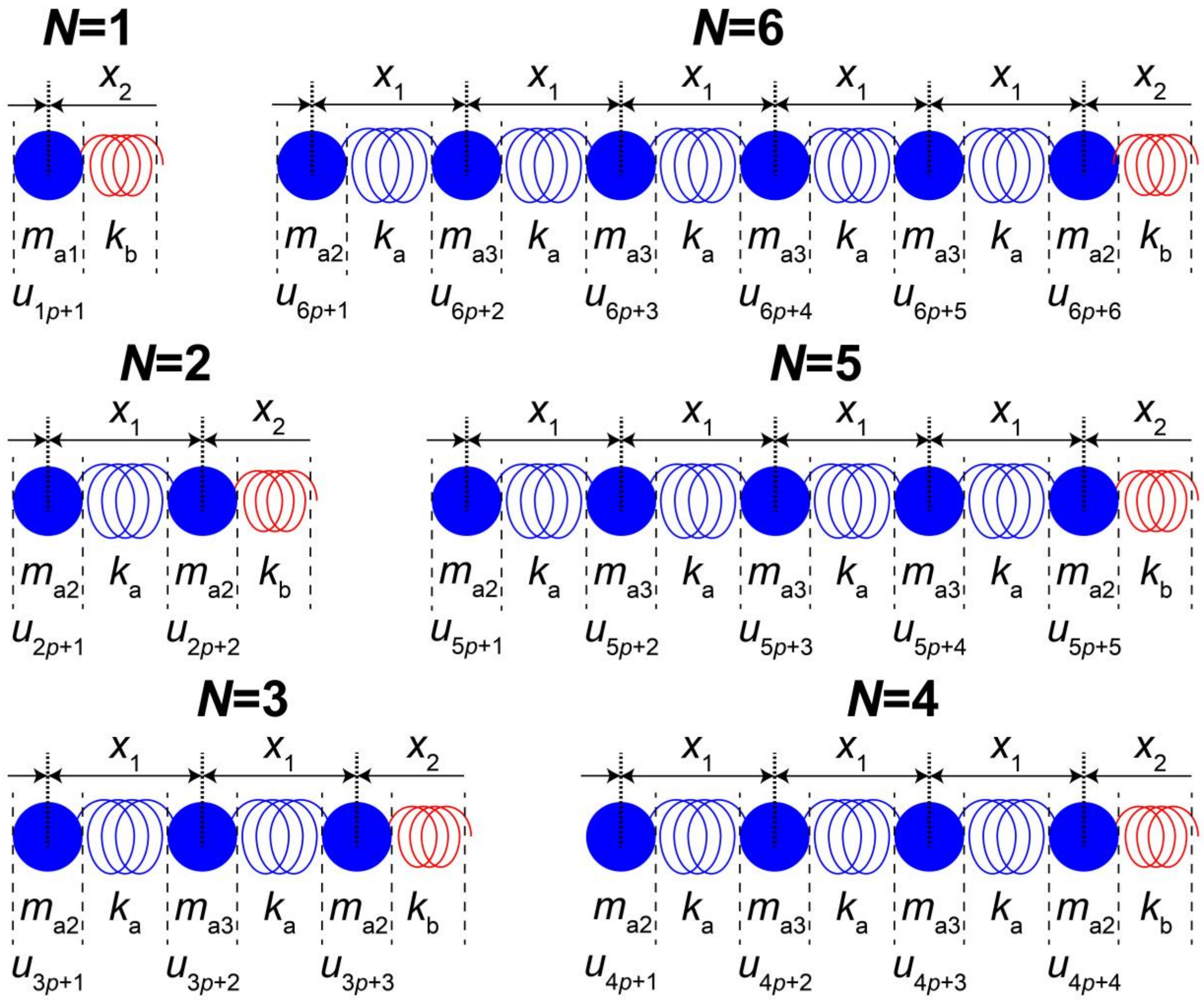

Supplementary Figure 13. Schematic drawing of the bead-spring model for *N*=1 to 6. Each Pb-I octahedron is coarse-grained by a blue bead; those Pb-I octahedra in direct contact are connected by the blue springs with a spring constant of k_a; those Pb-I octahedra separated by an organic layer are linked by the red springs with a spring constant of k_b.

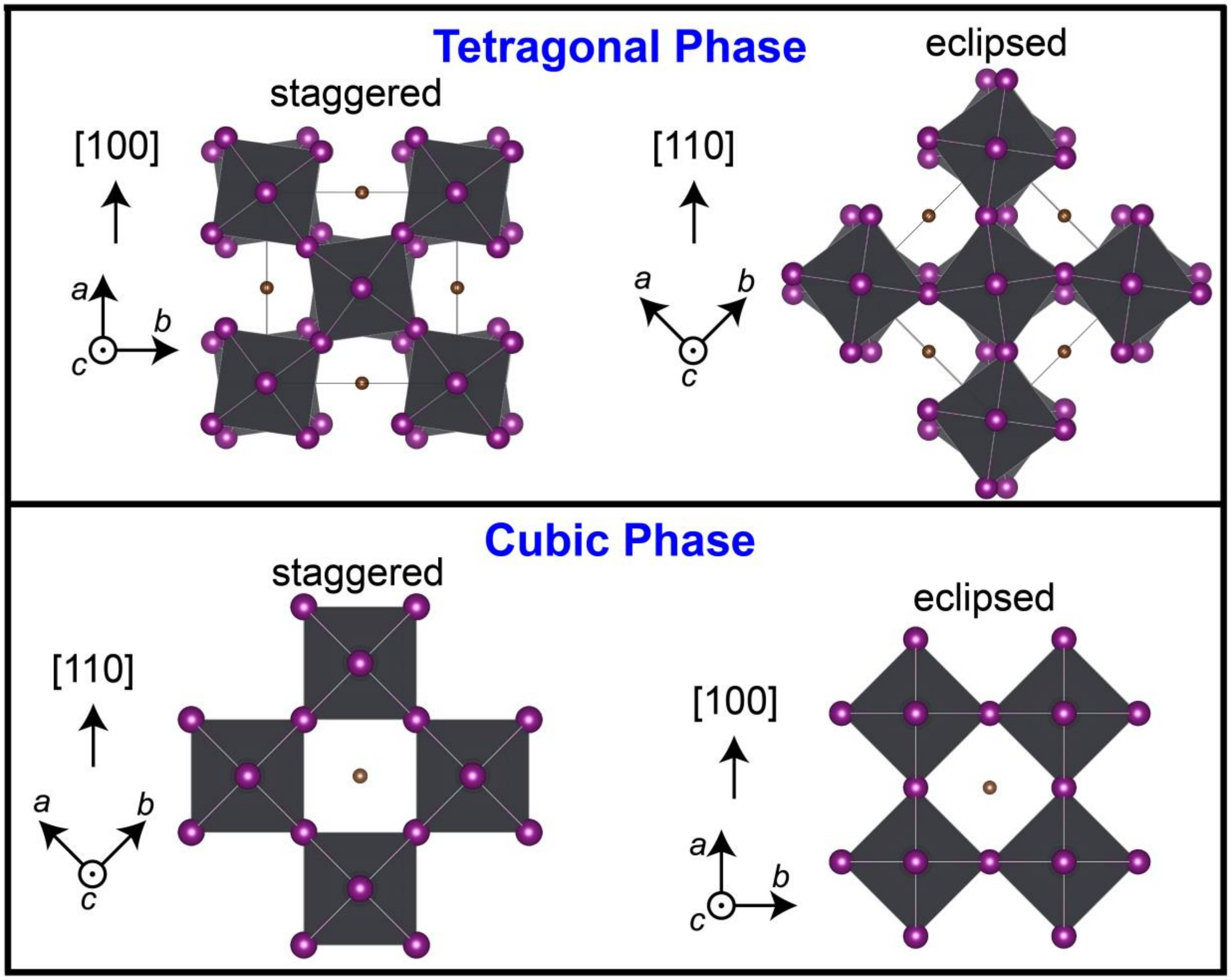

Supplementary Figure 14. Crystal structures and crystallographic orientations of the tetragonal and cubic phases of $MAPbI_3$. The octahedral layers along the [100] direction of the tetragonal phase, and [110] direction of the cubic phase, are stacked in a staggered manner. The octahedral layers along the [110] direction of the tetragonal phase, and [100] direction of the cubic phase, are stacked in an eclipsed manner. The stacking configuration in 2D-RPs follows the eclipsed manner.

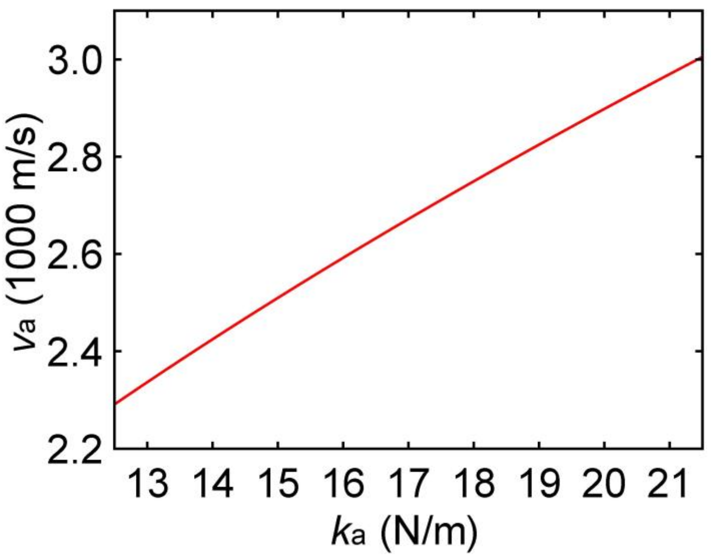

Supplementary Figure 15. Dependence of v_a on k_a for a single chain of $MAPbI_3$ beads. Each bead has a mass of m_{a3}=1.0295×10^{-24} kg and a spring length of 6.39 Å.

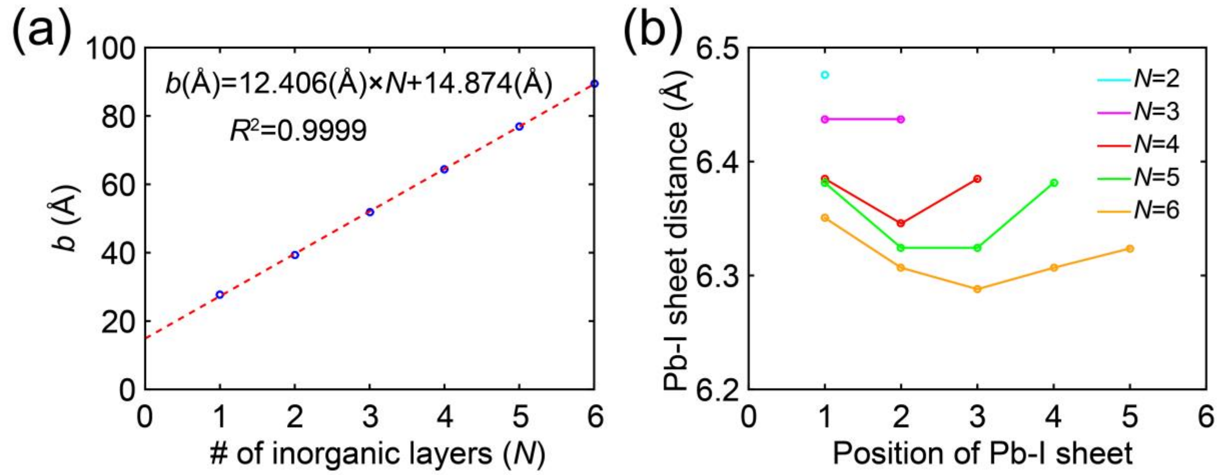

Supplementary Figure 16. (a) Dependence of *b* (the cross-plane lattice constant) on *N*, and its linear fit. (b) The distances between neighbouring Pb-I octahedral sheets in *N*=2 to 6.

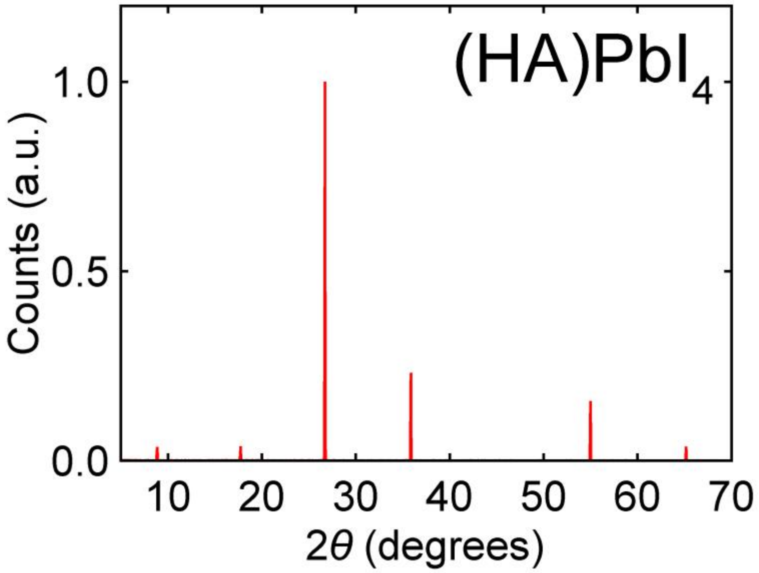

Supplementary Figure 17. Specular *θ*-2*θ* X-ray diffraction pattern of a single-crystal flake of $(HA)PbI_4$.

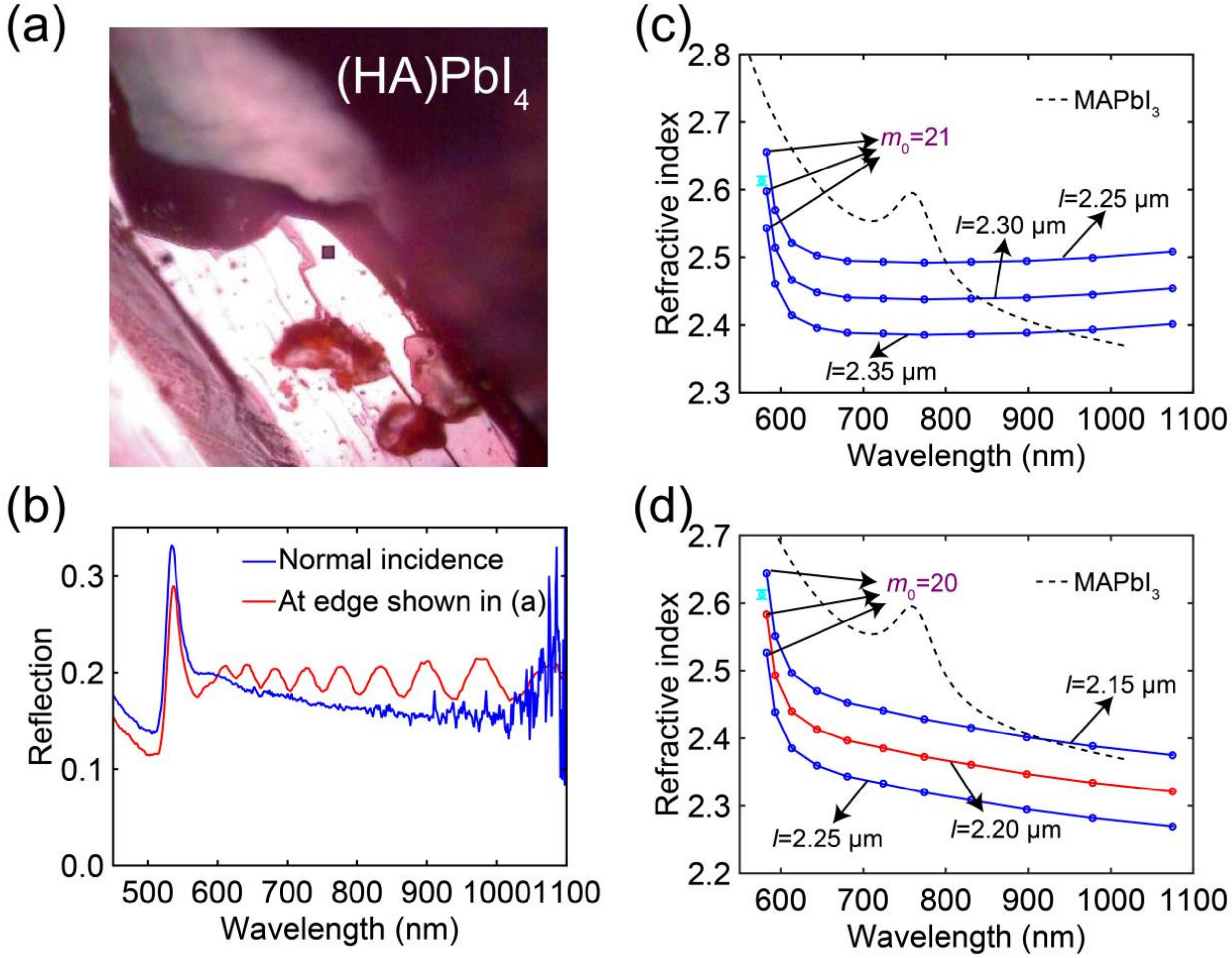

Supplementary Figure 18. Quantification of the refractive index for (HA)PbI4. (a) Optical microscopic image of an (HA)PbI4 flake. (b) Red: reflection of a thin layer of (HA)PbI4 taken at the edge of the flake as shown in (a); blue: reflection of (HA)PbI4 with the correct absolute amplitude (taken at exact normal incidence). (c) Several possible $n'(\lambda)$ curves obtained using the same trial mode number (m_0=21) and different trial sample thicknesses. (d) Several possible $n'(\lambda)$ curves obtained using the same trial mode number (m_0=20) and different trial sample thicknesses, with the correct $n'(\lambda)$ curve shown in red. (c) and (d) were calculated based on the interference pattern exhibited in the red curve in (b). The cyan dots in (c) and (d) denote the refractive index of (HA)PbI4 at the absorption edge determined from the blue curve in (b).

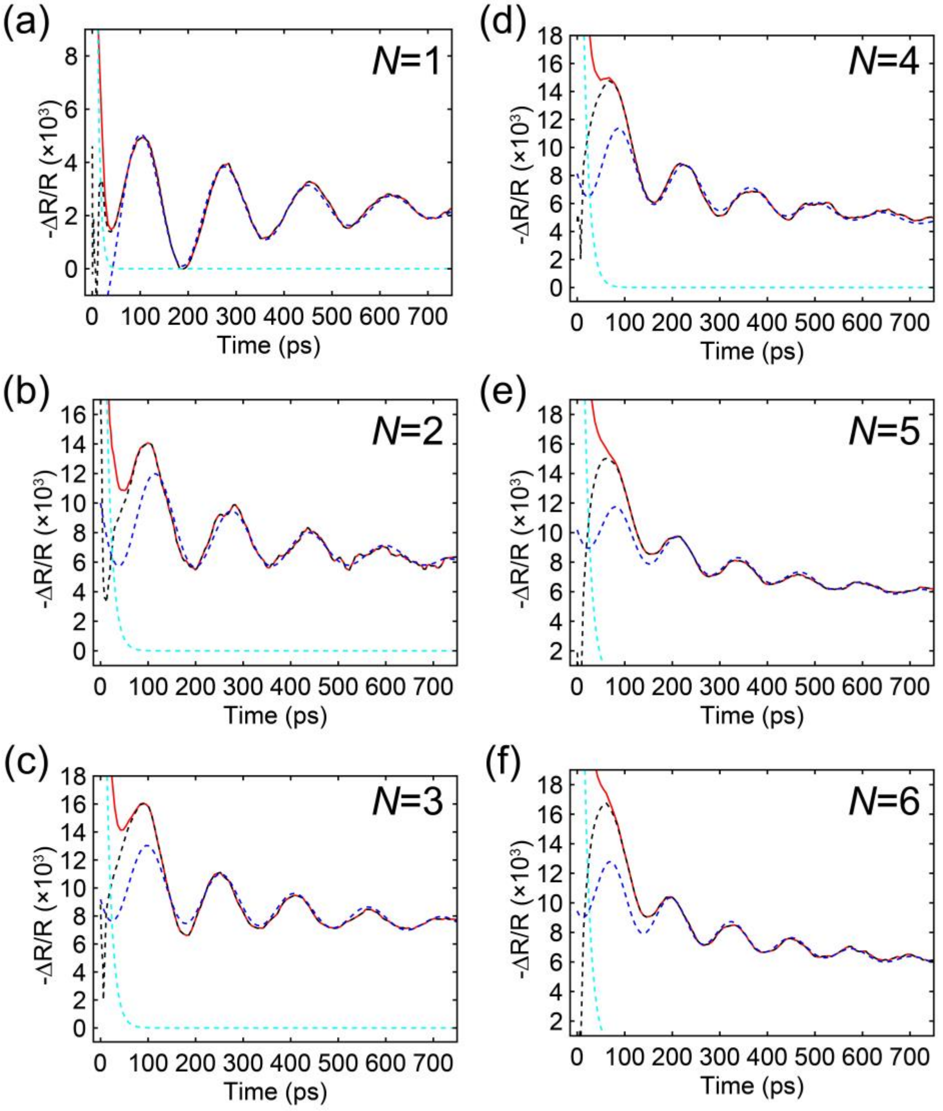

Supplementary Figure 19. Representative experimental and fitted kinetic traces of -Δ*R*/*R* at 955 nm for *N*=1 to 6. Red-solid lines are the experimental data; cyan-dashed lines have the form of $\mathrm{A}e^{-t/\tau_0}$ (representing hot carrier relaxation and 3rd order carrier recombination), which were subtracted from the experimental data and result in the black-dashed lines. The blue-dashed lines are best fits to the black-dashed lines and have the form $\mathrm{B}e^{-t/\tau_1} + \mathrm{C}e^{-t/\tau_2} + \mathrm{D}e^{-t/\tau_3}\sin\left(\frac{2\pi t}{T} - \varphi\right)$. Here $\mathrm{B}e^{-t/\tau_1}$ captures the 2nd order recombination, $\mathrm{C}e^{-t/\tau_2}$ represents the 1st order recombination and lattice temperature decay, $\mathrm{D}e^{-t/\tau_3}$ describes the decay of the amplitude of the sinusoidal oscillation. The oscillation period is denoted as T. An initial phase lag of φ is introduced to the sine function, to account for the fact that acoustic phonons are not instantaneously launched by the pump.

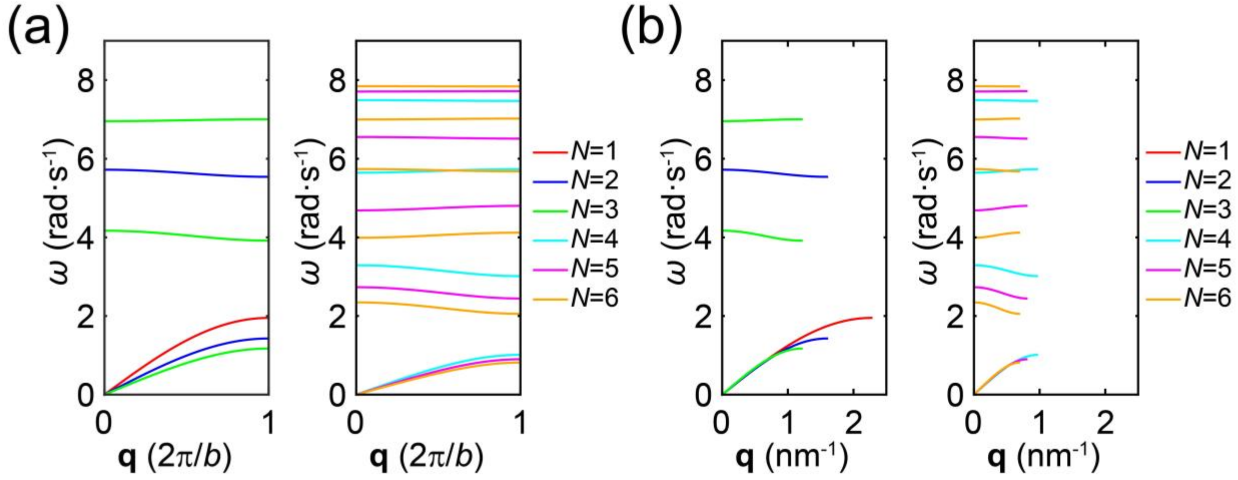

Supplementary Figure 20. Phonon dispersion diagrams calculated for the bead-spring model for *N*=1 to 6. k_a=17 N·m^{-1} and k_b=1.13 N·m^{-1} were used in the calculation. The wave vector **q** is plotted in unit of 2π/*b* in (a), and in unit of nm^{-1} in (b).

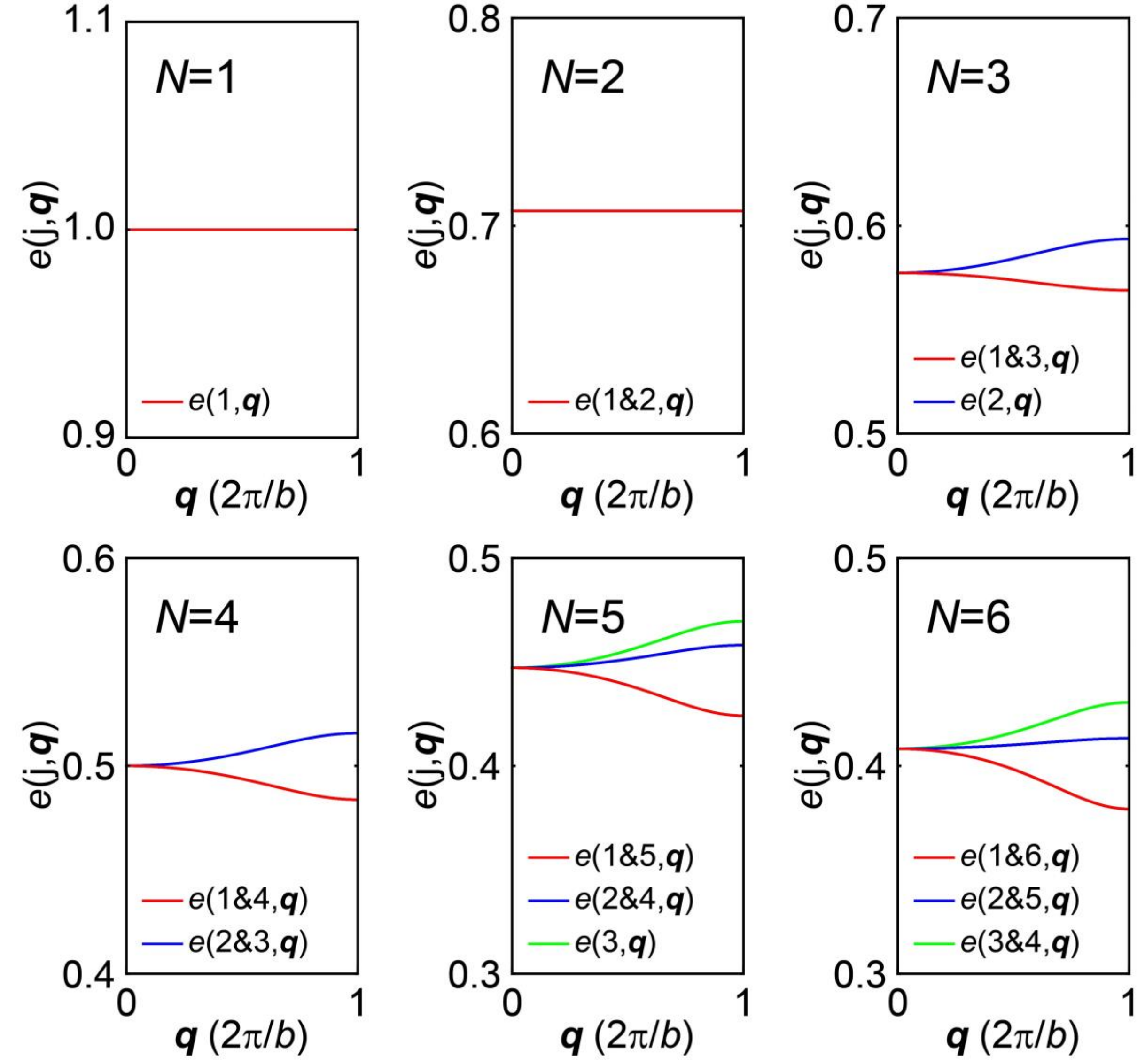

Supplementary Figure 21. The calculated eigen-displacements of the individual beads in a unit cell for *N*=1 to 6. The bead indices follow those defined in Supplementary Fig. 13. k_a=17 N·m^{-1} and k_b=1.13 N·m^{-1} were used in the calculation. Note that the polarization vector $\boldsymbol{e}(\mathbf{q}) = \begin{bmatrix} e(1,\mathbf{q}) \\ \dots \\ e(n,\mathbf{q}) \end{bmatrix}$ has length of unity.

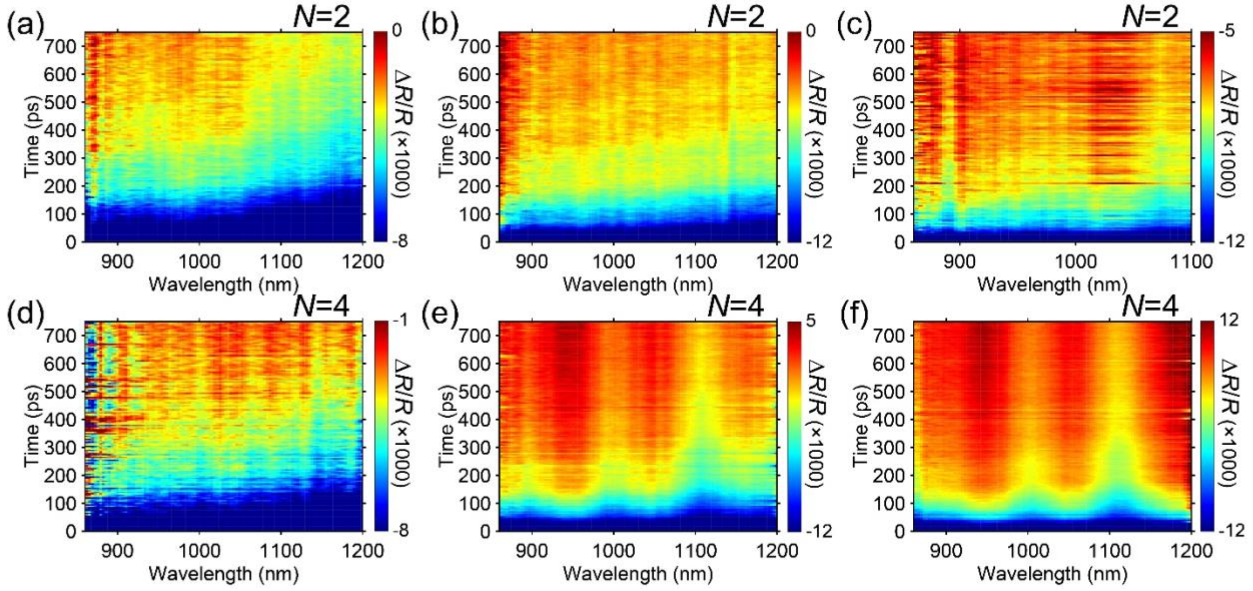

Supplementary Figure 22. Transient reflection spectral maps of *N*=2 and *N*=4 acquired with near-exciton gap pump excitation. (a) to (c) Δ*R*/*R* spectral maps for *N*=2 acquired with 570-nm pump excitation. Fluences used were 0.16, 0.41, and 0.82 mJ·cm^{-2}, respectively. (d) to (f) Δ*R*/*R* spectral maps for *N*=4 acquired with 640-nm pump excitation. Fluences used were 0.09, 0.18, and 0.26 mJ·cm^{-2}, respectively.

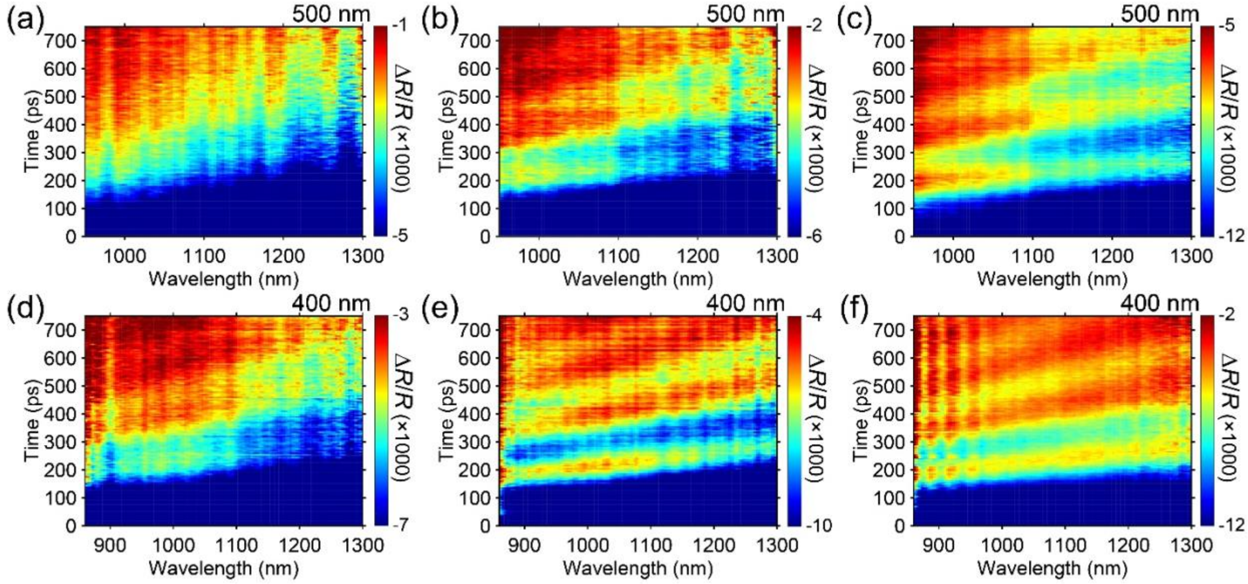

Supplementary Figure 23. Transient reflection measurements of *N*=2 with different pump wavelengths. (a) to (c) Δ*R*/*R* spectral maps acquired with 500-nm pump excitation. Fluences used were 0.05, 0.18, and 0.29 mJ·cm^{-2}, respectively. (d) to (f) Δ*R*/*R* spectral maps acquired with 400-nm pump excitation. Fluences used were 0.11, 0.21, and 0.35 mJ·cm^{-2}, respectively.

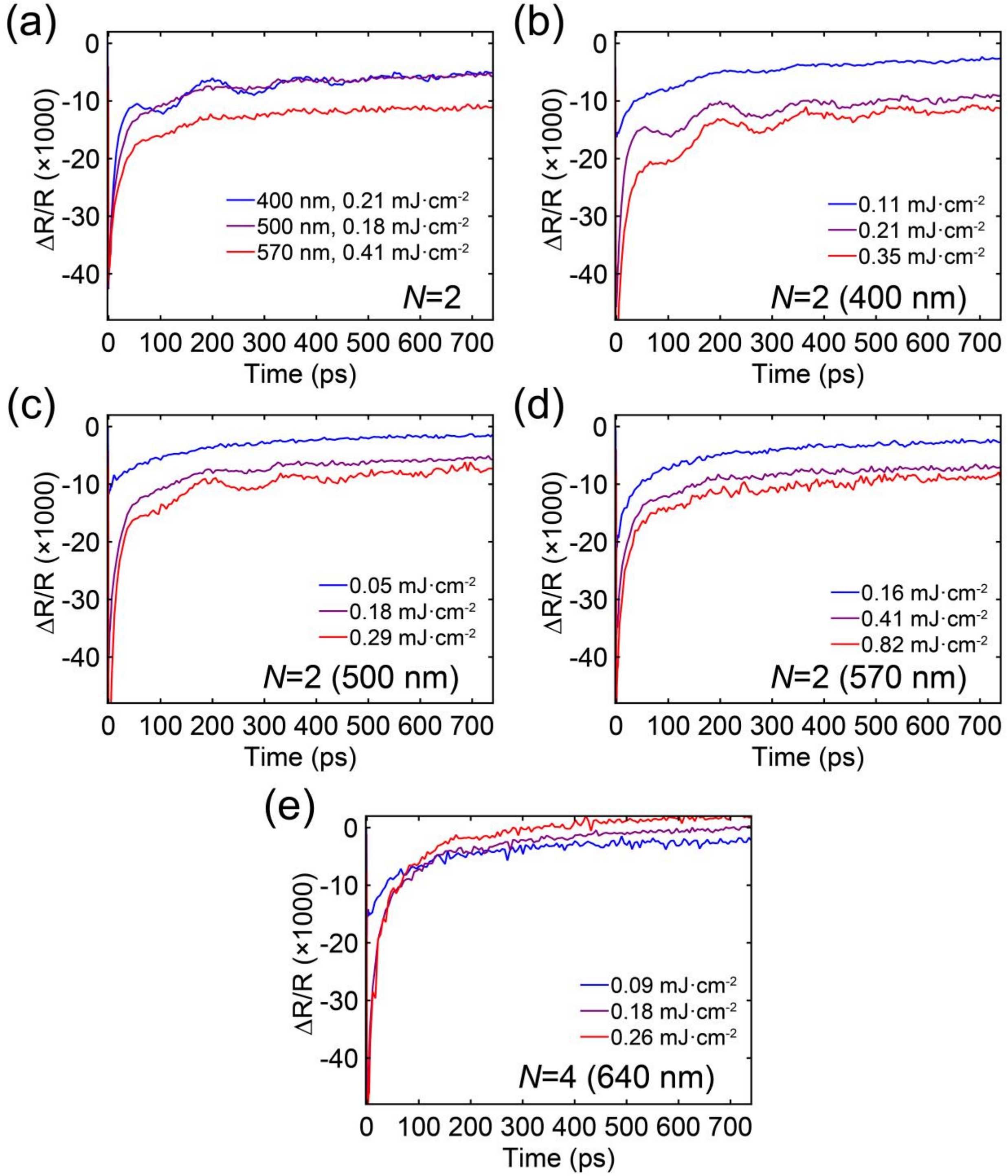

Supplementary Figure 24. Representative Δ*R*/*R* kinetics of *N*=2 and *N*=4 with different pump wavelengths and fluences. (a) Δ*R*/*R* kinetics of *N*=2 with different pump wavelengths. (b) Δ*R*/*R* kinetics of *N*=2 excited with 400-nm pump. (c) Δ*R*/*R* kinetics of *N*=2 excited with 500-nm pump. (d) Δ*R*/*R* kinetics of *N*=2 excited with 570-nm pump. (e) Δ*R*/*R* kinetics of *N*=4 excited with 640-nm pump.

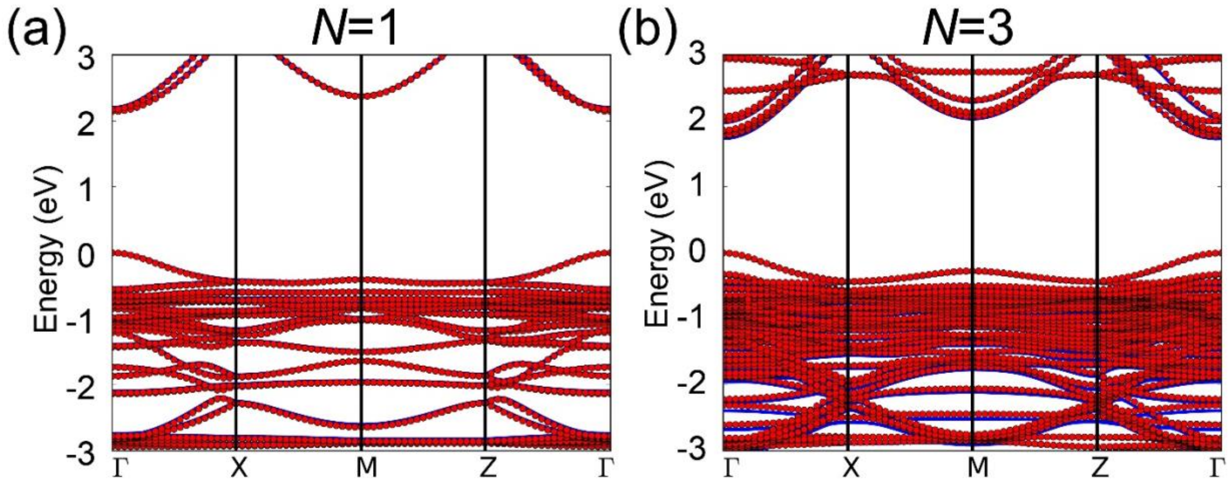

Supplementary Figure 25. Calculated electronic band structures for *N*=1 in (a) and *N*=3 in (b). Un-strained results are shown in blue circles; 1% strained results are shown in red circles. The calculated bandgap for *N*=1 is 2.15 eV (unstrained) and 2.14 eV (strained); the calculated bandgap for *N*=3 is 1.73 eV (unstrained) and 1.77 eV (strained).

	b (Å)	index of the strongest diffraction peak	2θ value (°)
N=1	27.652	(0 8 0)	25.7474
N=2	39.348	(0 12 0)	27.1696
N=3	51.960	(0 16 0)	27.4258
N=4	64.384	(0 20 0)	27.687
N=5	77.014	(0 24 0)	27.7766
N=6	89.408	(0 28 0)	27.9881

Supplementary Table 1. Lattice constants along *b* (cross-plane direction), indices of the strongest diffraction peak, and the corresponding 2*θ* values (with Cu-Kα radiation).

Supplementary Note 1. Two-step process to determine the below-bandgap $n'(\lambda)$

Step 1: determining several possible n'(λ) curves for each composition

We acquired static reflection spectra for several specific regions of each layered perovskite composition using a Filmetrics F40 microscope, and located the wavelengths of the reflection minima, from which we obtained several possible $n'(\lambda)$ curves (as those shown in Fig. 3d) using the equation $2n'(\lambda)l = m\lambda$, only one of which is correct. Note that destructive interferences occur when differences in the optical pathlength are equal to wavelength multiplied by an integer, since light reflected at the top interface (from a low- to a high-index medium) experiences an additional phase shift of π. Here the thickness l is an input parameter that was determined from SEM measurements, and m is a mode number (specific for each reflection minimum). Because only the edges can be side-viewed under SEM for precise thickness determination, we searched for flakes near the edges and recorded reflection spectra in those locations. Flakes of several-µm thickness were chosen, as still thicker flakes lead to unresolved reflection minima (too closely-spaced, especially on the blue side where λ is small), whereas thinner flakes (in the 1~3 µm range) produce undesirably sparse spacings of reflection minima and hence too few (poorly constraining) data points on the $n'(\lambda)$ curve. Note that in transient reflection measurements for the study of CLAPs, we purposefully examined large flakes with lateral dimensions in the millimeter range. However, these laterally large flakes are also thick in the cross-plane dimension (in the tens-of-µm range), thus they do not exhibit resolvable reflection minima. As a result, for this step of index determination, we did not use large flakes but instead examined small flakes, which have extruded, thinner regions at the edges.

Supplementary Figs. 2 to 5 present the SEM images and reflection spectra of thin regions at the edges, and several possible $n'(\lambda)$ curves for N=5, 4, 3 and 2. Similar results for N=6 are shown in Fig. 3 of the main text. Note that for N=6 to 2, we could examine a-few-µm-thick extrusions at the edges. However, the N=1 flakes tend to grow thicker so we couldn't locate a clean, thin extrusion (as we could for N=2 to 6). Nevertheless, as presented in Supplementary Fig. 6a, occasionally we could find fissures inside the N=1 flakes, which are parallel to the Pb-I sheets and create clean interfaces. These interfaces reflect light and yield interference pattern in the reflection spectra (when measured from the top surface) of similar quality as for N=2 to 6 flakes. Because of the different reflective properties of these fissures, these regions produce

distinct contrast when viewed from the top surface (Supplementary Fig. 7a), so they can be identified and measured under the Filmetrics microscope (Supplementary Fig. 7b).

Most fissures do not extend to the crystal edge, but those reaching the edges of the flake (which were only rarely seen) permit the thickness measurements. A 12.3-μm thick layer, due to a fissure formed near and extended to the crystal edge, is shown in Supplementary Figs. 6a and 6b. Because of the large layer thickness, the reflection minima on the blue side of the spectrum cannot be well resolved, thereby prohibiting the determination of $n'(\lambda)$ (see the green region in Supplementary Figs. 6c and 6d). Nonetheless, a thin layer with an unknown thickness terminated by a fissure formed inside the flake (which does not extend to the edge of the flake), manifested as the brighter region at the centre of the optical microscopic image shown in Supplementary Fig. 7a, exhibits less dense, and hence identifiable reflection minima throughout the entire below bandgap region (Supplementary Fig. 7b). Because the thick layer with a known thickness and the thin layer with an unknown thickness have the same $n'(\lambda)$, we set the thickness of the thin layer as a fitting parameter, which can be determined by matching the $n'(\lambda)$ curves calculated from both layers, as displayed in Supplementary Fig. 7c. Using the fitted thickness (4.86 μm) for the thin layer, we could then obtain several possible $n'(\lambda)$, as further plotted in Supplementary Fig. 7d. Note that an incorrect thickness of the thin layer will lead to disagreements between the $n'(\lambda)$ dispersions obtained from the two layers for *all* the trial mode numbers used in the calculations. For example, we plotted the calculated $n'(\lambda)$ curves for the thin layer with a thickness of 4.96 μm (Supplementary Fig. 7e) and 4.76 μm (Supplementary Fig. 7f), which are only 2% off from the true thickness (4.86 μm); such incorrect thickness leads to disagreement in the slopes of $n'(\lambda)$ between the two sets of curves (for the thick and the thin flakes).

Step 2: determining n′(λ) at the absorption edge

In step 1, the spectral locations of the reflection minima vary negligibly with small deviations of the incidence angle from normal incidence at 0°. This was evident from the fact that reflection spectra collected from the two opposite sides of the flakes exhibit identical wavelengths of reflection minima (note that depending on the smoothness, incidence angles from the two sides may not be the same, especially for the small flakes used in step 1). Transfer matrix calculation demonstrates that the shifts of reflection minima below 10° incidence angle are no more than 10 nm compared to the normal incidence case (see Supplementary Fig. 9); this arises because at

small θ values, the additional factor of cosθ introduced into the equation $2n'(\lambda)l = m\lambda$ (which then becomes $2n'(\lambda)l \cdot \cos\theta = m\lambda$) is very close to 1; here θ is the angle of refraction, which is notably still smaller than the incidence angle due to the Snell's law.

Although the reflection minima wavelengths are not sensitive to small deviations of the incidence angle from 0°, the absolute reflection intensities are. This arises because at oblique incidence the beam is deflected by the sample away from the microscope objective, which reduces the collection efficiency. To get the absolute reflectance for the quantification of $n'(\lambda)$ at some wavelength (the specific choice of wavelength is discussed later), we examined large flakes in the centre regions with good surface smoothness at normal incidence. Since for these large flakes we could not measure the thickness (in the centre regions), the phase relation between the top- and bottom-reflected waves in the below bandgap regime is unknown, and therefore we did not use the absolute reflectance in this region to deduce $n'(\lambda)$. However, at the wavelength of the absorption edge (denoted as λ_{edge}) where the material becomes absorptive, the wave cannot reach the bottom of the flake due to attenuation, therefore the reflection minima arising from interference effect disappear. To illustrate this point, we show in Supplementary Fig. 10a the transfer-matrix calculated reflectivity of a 6-μm thick slab with various real and imaginary parts of the refractive index, which are denoted as n' and n'', respectively. The oscillatory change of reflectivity with n' observed at small n'' values (specifically, when n''<0.5) shown in Supplementary Fig. 10a resembles the oscillatory change of reflectivity with wavelength in the below bandgap range, as both arise from the interference effects governed by the same equation. With n'' larger than 0.5, light does not reach the bottom of the flake, so no interference effect is present. This is further demonstrated in Supplementary Fig. 10b, which is the transmission analogue of Supplementary Fig. 10a and shows that transmission at n''>0.5 is indeed negligibly small. Note that 0.5 here is only an approximate boundary for n'' and need not be precise. As a result, we can conclude that at λ_{edge} where interference effect just disappears, n'' should be equal or greater 0.5. We set an upper bound of 1.0 on n'' at λ_{edge} for the reasons that (i) n'' has to vary continuously; (ii) λ_{edge} is less than 10 nm bluer than the bluest reflection minimum arising from interference effect (as shown in Supplementary Figs. 2 to 7, and Fig. 3); n'' cannot increase from 0.5 to a value larger than 1.0 with such small wavelength range (Supplementary Fig. 11 shows that the transmission at λ_{edge} is either above or close to zero).

Note that the reflection at λ_{edge} also exhibits a minimum (equivalently, we can also say that this minimum in reflection is how λ_{edge} is defined), for the following two reasons: (1) On the blue side of λ_{edge}, both n'' and n' increase with a decreasing wavelength (a universal trend for all direct-bandgap semiconductors), which, as evident from Supplementary Fig. 10a, leads to an increase of the reflectivity; (2) On the red side of λ_{edge}, both n'' and n' decrease, which should decrease the reflectivity. On the red part of the below bandgap region (a few hundred nm away from λ_{edge}), n'' is nearly 0 (evident from 70% to 90% transmission measured for a-few-tens-μm thick flakes; see Supplementary Fig. 11). Therefore, on the red side of λ_{edge}, n'' has to gradually decrease to zero, which is manifested by the growing amplitude of the oscillations with an increasing wavelength in the reflection spectra (Supplementary Figs. 2 to 7, and Fig. 3). However, on the red side of λ_{edge}, the reflection from the bottom interface starts to contribute; this results in an enhanced reflectivity with an increasing wavelength.

The measured reflection spectra with the correct absolute amplitude are plotted in Supplementary Fig. 11. Here each λ_{edge} is highlighted by a vertical, black-dashed line, where a minimum in reflection appears. This reflection minimum at λ_{edge} can be distinguished from those resulting from the interference effects (should the latter arise in the reflection spectrum), because on the blue side of λ_{edge} the reflectivity increases very slowly with a decreasing wavelength, whereas reflection minima due to interference effects have very close spacings as the wavelength decreases.

As discussed above, we confined n'' between 0.5 to 1.0, and found the corresponding n', for each n'', that gives a calculated reflectivity matching the measured reflection at λ_{edge}. The identified Re(n) as a function of n'' for N=1 to 6 are shown in Supplementary Fig. 10c, which are further plotted in Supplementary Fig. 10d as a function of wavelength for different compositions. The error bars in Supplementary Fig. 10d correspond to uncertainties in n' shown in Supplementary Fig. 10c, which in turn arise from the uncertainties in n''. Knowing n' at λ_{edge} (shown as those cyan dots in Supplementary Figs. 2 to 7, and Fig. 3) permits the identification of the correct $n'(\lambda)$ curve (shown in red) from the set of curves. Also note that for the determination of n' when n''>0.5, the actual layer thickness is not important, because the wave is strongly attenuated and no contribution from the bottom-side reflection is present.

Supplementary Note 2. Bead-spring model

Supplementary Fig. 13 shows schematics of the bead-spring model. We differentiated the masses for 1) an *isolated* octahedron (with the chemical formula of PbI_4) as appearing in *N*=1 with m_{a1}=1.187×10^{-24} kg; (2) an *edge*-octahedron (with the chemical formula of $(MA)_{0.5}PbI_{3.5}$) as appearing in *N*=2~6 with m_{a2}=1.1082×10^{-24} kg; (3) an *inner* octahedron (with the chemical formula of $MAPbI_3$) as appearing in *N*=2~6, ∞, with m_{a3}=1.0295×10^{-24} kg. The bead-spring model is in one-dimension only, so the words "isolated", "edge" and "inner" are only related to the cross-plane direction.

As an example, for *N*=3, the equations of motion are written as

$$m_{a2}\ddot{u}_{3p+1} = k_a\left(u_{3p+2} - u_{3p+1}\right) - k_b\left(u_{3p+1} - u_{3p}\right)$$

$$m_{a3}\ddot{u}_{3p+2} = k_a\left(u_{3p+3} - u_{3p+2}\right) - k_a\left(u_{3p+2} - u_{3p+1}\right)$$

$$m_{a2}\ddot{u}_{3p+3} = k_b\left(u_{3(p+1)+1} - u_{3p+3}\right) - k_a\left(u_{3p+3} - u_{3p+2}\right)$$

Here p is the index of the unit cell. Assuming a solution of the form $u_{3p+j} = U_j\exp[ipl_3q - i\omega t]$, where q is the wavevector, $j = 1, 2, 3$, and $l_3 = 2x_1 + x_2$ is equal to the lattice parameter along b, we obtain the following set of equations,

$$m_{a2}\omega^2U_1 - k_aU_1 - k_bU_1 + k_aU_2 + k_bU_3\exp(-il_3q) = 0$$

$$m_{a3}\omega^2U_2 + k_aU_1 - k_aU_2 - k_aU_2 + k_aU_3 = 0$$

$$k_bU_1\exp(il_3q) + k_aU_2 - k_aU_3 - k_bU_3 + m_{a2}\omega^2U_3 = 0$$

Non-trivial solution exists only if the determinant of the coefficients of U_j is zero, i.e.,

$$\begin{vmatrix} m_{a2}\omega^2 - k_a - k_b & k_a & k_b\exp(-il_3q) \\ k_a & m_{a3}\omega^2 - 2k_a & k_a \\ k_b\exp(il_3q) & k_a & m_{a2}\omega^2 - k_a - k_b \end{vmatrix} = 0$$

Similarly, the dispersion equation for *N*=1 is $m_{a1}\omega^2 - 2k_b + k_b[\exp(-il_1q) + \exp(il_1q)] = 0$.

The dispersion matrix for *N*=2 is

$$\begin{vmatrix} m_{a2}\omega^2 - k_a - k_b & k_a + k_b\exp(-il_2q) \\ k_a + k_b\exp(il_2q) & m_{a2}\omega^2 - k_a - k_b \end{vmatrix} = 0$$

The dispersion matrix for *N*=4 is

$$\begin{vmatrix} m_{a2}\omega^2 - k_a - k_b & k_a & 0 & k_b \exp(-il_4 q) \\ k_a & m_{a3}\omega^2 - 2k_a & k_a & 0 \\ 0 & k_a & m_{a3}\omega^2 - 2k_a & k_a \\ k_b \exp(il_4 q) & 0 & k_a & m_{a2}\omega^2 - k_a - k_b \end{vmatrix} = 0$$

The dispersion matrix for N=5 is

$$\begin{vmatrix} m_{a2}\omega^2 - k_a - k_b & k_a & 0 & 0 & k_b \exp(-il_5 q) \\ k_a & m_{a3}\omega^2 - 2k_a & k_a & 0 & 0 \\ 0 & k_a & m_{a3}\omega^2 - 2k_a & k_a & 0 \\ 0 & 0 & k_a & m_{a3}\omega^2 - 2k_a & k_a \\ k_b \exp(il_5 q) & 0 & 0 & k_a & m_{a2}\omega^2 - k_a - k_b \end{vmatrix} = 0$$

The dispersion matrix for N=6 is

$$\begin{vmatrix} m_{a2}\omega^2 - k_a - k_b & k_a & 0 & 0 & 0 & k_b \exp(-il_6 q) \\ k_a & m_{a3}\omega^2 - 2k_a & k_a & 0 & 0 & 0 \\ 0 & k_a & m_{a3}\omega^2 - 2k_a & k_a & 0 & 0 \\ 0 & 0 & k_a & m_{a3}\omega^2 - 2k_a & k_a & 0 \\ 0 & 0 & 0 & k_a & m_{a3}\omega^2 - 2k_a & k_a \\ k_b \exp(il_6 q) & 0 & 0 & 0 & k_a & m_{a2}\omega^2 - k_a - k_b \end{vmatrix} = 0$$

Supplementary Note 3. Determination of the below-bandgap $n'(\lambda)$ for (HA)PbI_4

The (HA)PbI_4 single-crystal flakes (XRD pattern shown in Supplementary Fig. 17) exhibit interference patterns in the reflection spectrum (Supplementary Fig. 18) due to fissure formed inside the crystal. However, we could not locate a fissure extended to the edge of the crystal, as we could for N=1, for the determination of the corresponding thickness. Here we used a slightly different approach to obtain $n'(\lambda)$ for (HA)PbI_4. We first determined $n'(\lambda)$ at the absorption edge as described in Supplementary Note 1, step 2. The determined $n'(\lambda)$ at the absorption edge is shown as the cyan dot in Supplementary Figs. 18c and 18d. We then recall that, as discussed in the main text, $T(\lambda) = \frac{\lambda}{2vn'(\lambda)}$, or equivalently, $v = \frac{\lambda}{2T(\lambda)n'(\lambda)}$. Because the velocity is fixed, we have $\frac{\lambda_1}{2T(\lambda_1)n'(\lambda_1)} = \frac{\lambda_2}{2T(\lambda_2)n'(\lambda_2)}$, or $\frac{n'(\lambda_2)}{n'(\lambda_1)} = \frac{\lambda_2 T(\lambda_1)}{\lambda_1 T(\lambda_2)}$, where λ_1 and λ_2 are any two wavelengths in the probed spectral window shown in Fig. 5a. Since $\frac{\lambda_2 T(\lambda_1)}{\lambda_1 T(\lambda_2)}$ can be obtained from the transient spectral map, the equation $\frac{n'(\lambda_2)}{n'(\lambda_1)} = \frac{\lambda_2 T(\lambda_1)}{\lambda_1 T(\lambda_2)}$ provides a constraint on the shape of the $n'(\lambda)$ curve. As shown in Supplementary Fig. 18c, we first varied the value of m_0 (the mode number of the first reflection dip), and found that the shape of $n'(\lambda)$ cannot match with that predicted by the equation $\frac{n'(\lambda_2)}{n'(\lambda_1)} = \frac{\lambda_2 T(\lambda_1)}{\lambda_1 T(\lambda_2)}$, regardless of any trial thickness. Only with the correct m_0, as shown in Supplementary Fig. 18d, could we obtain the $n'(\lambda)$ curve that both match with the equation $\frac{n'(\lambda_2)}{n'(\lambda_1)} = \frac{\lambda_2 T(\lambda_1)}{\lambda_1 T(\lambda_2)}$, and the cyan dot. The correct $n'(\lambda)$ curve for (HA)PbI_4 is shown in Supplementary Fig. 18d as the red line.

Supplementary Note 4. Calculation of the mean square displacement

The (time-averaged) mean square displacements of a bead in the bead-spring model is calculated as $\langle |u(j,t)|^2 \rangle = \frac{\hbar}{2Nm_j} \sum_{\mathbf{q}} [\omega(\mathbf{q})]^{-1} \, [1 + 2n(\mathbf{q},T)] |e(j,\mathbf{q})|^2$, where *u* stands for the displacement (see Supplementary Fig. 13), *j* labels the jth bead in a unit cell, $\omega(\mathbf{q})$ is the angular frequency at wavevector **q**, $e(j,\mathbf{q})$ is the jth component of the polarization vector, $n(\mathbf{q},T)$ is the phonon occupation number. For phonons we have $n(\mathbf{q},T) = \frac{1}{\exp[\hbar\omega(\mathbf{q})/k_B T]-1}$ with k_B being the Boltzmann constant and *T* being the temperature. The computed dispersion diagrams and the $e(j,\mathbf{q})$ of each individual bead are plotted in Supplementary Figs. 20 and 21, respectively. Note that in Supplementary Figs. 20 and 21, the maximum of **q** is $2\pi/b$ instead of π/b, because the unit cell length of the bead-spring model is half of that of the actual material's unit cell; the latter is denoted as *b*. The pump pulse raises the temperature, and with it the mean square displacement via the change of $n(\mathbf{q},T)$, which is the only temperature dependent term. At small ΔT, the response of the mean square displacement to the temperature rise is written as $\frac{\Delta\langle |u(jl,t)|^2 \rangle}{\Delta T} = \frac{\hbar}{2Nm_j} \sum_{\mathbf{q}} [\omega(\mathbf{q})]^{-1} \left[1 + 2\frac{\Delta n(\mathbf{q},T)}{\Delta T}\right] |e(j,\mathbf{q})|^2$. The mean absolute displacement $\Delta\langle |u(t)| \rangle$ plotted in Fig. 5e is taken as the average value of $\Delta\langle |u(jl,t)| \rangle$ of all the individual beads in a unit cell. The base temperature used in the calculation was 298 K. Qualitatively, the *N* dependence of $\Delta\langle |u(t)| \rangle$ arises from the different number densities of Pb-I octahedra. Upon the injection of the same amount of energy, lower members with smaller number densities of octahedra experience stronger vibration amplitudes as dictated by energy and momentum conservation.

Because only the acoustic phonons are responsible for the probe oscillations, the summation over **q** was performed for the acoustic branch only. We further assumed that acoustic phonons are only populated within a small $\Delta\omega(\mathbf{q})$ range of 0.1×10^{12} rad·s^{-1} wide near the Γ point, for the reason below.[1] Assuming a skin depth of 100 nm (for the optical pump) and a sound velocity of 1600 m·s^{-1}, the temporal duration of the excited acoustic phonons is ~62.5 ps, which is converted to a frequency range of 0.1×10^{12} rad·s^{-1}. The same $\Delta\omega(\mathbf{q})$ range was used in all the calculations. We found variations of the spectral location and width of $\Delta\omega(\mathbf{q})$ do not alter the trend of $\Delta\langle |u(t)| \rangle$ plotted in Fig. 5e.

Both the thermoelastic and the deformation potential effects can be responsible for the generation of the CLAPs[1]. Thermoelastic effect relates impulsive heating of the lattice by hot carriers; these hot carriers (including both electrons and holes) quickly relax to the exciton energy though heat exchange with the lattice, which causes a lattice thermal expansion and with it CAPs. As to the deformation potential mechanism, strain is induced by the modification in energy of the electronic distribution, which in our case involves electrons and holes created by the above-exciton-gap pump. As shown in Supplementary Figs. 22, 23 and 24, our additional transient reflection measurements show that thermoelastic effect is mainly responsible for the CLAP signatures in the transient reflection spectral maps.

Supplementary References

1. Ruello, P., Gusev, V. E. Physical mechanisms of coherent acoustic phonons generation by ultrafast laser action. *Ultrasonics* **56**, 21-35 (2015).
